# A four-lncRNA risk signature for prognostic prediction of osteosarcoma

**DOI:** 10.3389/fgene.2022.1081478

**Published:** 2023-01-04

**Authors:** Huanlong Liu, Chao Chen, Long Liu, Zengtao Wang

**Affiliations:** ^1^ Hand and Foot Surgery, Shandong Provincial Hospital, Cheeloo College of Medicine, Shandong University, Jinan, China; ^2^ Hand and Foot Surgery, Shandong Provincial Hospital Affiliated to Shandong First Medical University, Jinan, China; ^3^ Engineering Research Center of Failure Analysis and Safety Assessment, Shandong Analysis and Test Center, Qilu University of Technology (Shandong Academy of Sciences), Jinan, China

**Keywords:** osteosarcoma, long noncoding RNA, risk signature, prognostic biomarker, bioinformatics analysis, TARGET database

## Abstract

**Aim:** Osteosarcoma is the most common primary malignant tumor of bone. However, our understanding of the prognostic indicators and the genetic mechanisms of the disease progression are still incomplete. The aim of this study was to identify a long noncoding RNA (lncRNA) risk signature for osteosarcoma survival prediction.

**Methods:** RNA sequencing data and relevant clinical information of osteosarcoma patients were downloaded from the database of Therapeutically Applicable Research to Generate Effective Treatments (TARGET). We analyzed the differentially expressed lncRNAs between deceased and living patients by univariate and multivariate Cox regression analysis to identify a risk signature. We calculated a prognostic risk score for each sample according to this prognosis signature, and divided patients into high-risk and low-risk groups according to the median value of the risk score (0.975). Kaplan–Meier analysis and receiver operating characteristic (ROC) curve statistics were used to evaluate the performance of the signature. Next, we analyzed the signature’s potential function through Gene Ontology (GO), Kyoto Encyclopedia of Genes and Genomes (KEGG), and gene-set enrichment analysis (GSEA). Lastly, qRT-PCR was used to validate the expression levels of the four lncRNAs in clinical samples.

**Results:** Twenty-six differentially expressed lncRNAs were identified between deceased and living patients. Four of these lncRNAs (CTB-4E7.1, RP11-553A10.1, RP11-24N18.1, and PVRL3-AS1) were identified as independent prognostic factors, and a risk signature of these four lncRNAs for osteosarcoma survival prediction was constructed. Kaplan–Meier analysis showed that the five-year survival time in high-risk and low-risk groups was 33.1% and 82.5%, and the area under the curve (AUC) of the ROC was 0.784, which demonstrated that the prognostic signature was reliable and had the potential to predict the survival of patients with osteosarcoma. The expression level of the four lncRNAs in osteosarcoma tissues and cells was determined by qRT-PCR. Functional enrichment analysis suggested that the signature might be related to osteosarcoma through regulation of the MAPK signaling pathway, the PI3K-Akt signaling pathway, and the extracellular matrix and also provided new insights into the study of osteosarcoma, including the role of papillomavirus infection, olfactory receptor activity, and olfactory transduction in osteosarcoma.

**Conclusion:** We constructed a novel lncRNA risk signature that served as an independent biomarker for predicting the prognosis of osteosarcoma patients.

## Introduction

As the most common malignant tumor of bone, osteosarcoma has a poor prognosis and a high incidence rate in children and adolescents (Kansara et al., 2014). In most cases, osteosarcomas can be found in the long bones of limbs, near the metaphyseal growth plate. Femur, tibia, and humerus are common sites, while skull, jaw, and pelvis are much less common (Ritter and Bielack, 2010; Simpson and Brown, 2018). Current therapies incorporate surgical resection with further combinational chemotherapy including doxorubicin, methotrexate, cisplatin, or ifosfamide, which not only shrinks the tumor but also eradicates micro-metastases and cures about 70% of patients (Lamoureux et al., 2007). However, for the past 30 years, the five-year survival rate of 20% for patients with metastatic or relapsed osteosarcoma has remained virtually unchanged (Collins et al., 2013). Despite the fact that it was known that tumor size, localized sites, present stage, and reaction to chemotherapy were of great significance in predicting overall survival, our perception and understanding of the prognostic indicators as well as the genetic mechanisms of disease progression were still incomplete and needed further exploration. It has been reported that genomic complexity and instability played a crucial role in the prognosis of osteosarcoma. Therefore, it was necessary to optimize the early detection, early diagnosis, and prognosis prediction of osteosarcoma from the perspective of molecular genetics.

Over the last decade, non-coding RNA has become a hot topic in research on diagnostic biomarkers and mechanism of many kinds of tumors. Long non-coding RNA (lncRNA) is a type of non-coding RNA with a length >200 nucleotides (Quinn and Chang, 2016), which has attracted worldwide attention because of its role in numerous diseases. Recent next-generation and high-throughput sequencing techniques have led to major breakthroughs in lncRNA identification and characterization. Accumulating evidence revealed that various lncRNAs were dysregulated in cancers, and were related to cancer recurrence, metastasis, and poor prognosis through epigenetic, transcriptional, and posttranscriptional mechanisms in various types of biological processes, including tumor initiation, growth, and metastasis (Kopp and Mendell, 2018; Sanchez Calle et al., 2018). Some studies have proved that lncRNAs were related to osteosarcoma progression (Wang et al., 2018; Chi et al., 2019; Zhu et al., 2019; Shen and Cheng, 2020). Nevertheless, whether lncRNAs could be used as prognostic markers of osteosarcoma was still unknown. Thus, further study of the clinical prognostic value of lncRNAs was definitely needed to offer osteosarcoma patients better therapeutic options. Existing and current studies were marshalled to categorize lncRNAs as robust predictors for osteosarcoma patients’ prognosis and to establish a prognostic risk signature based on comprehensive analysis of RNA sequencing data.

Our goal was to establish a prognostic model for osteosarcoma survival prediction, making use of RNA expression data as the foundation of a broad spectrum of genetic and clinical factors. A large cohort of osteosarcoma patients from the Therapeutically Applicable Research to Generate Effective Treatments (TARGET) project was used to identify potential lncRNAs related to the overall survival of patients with osteosarcoma. The lncRNA expression profiles and clinical characteristics were analyzed with univariable Cox regression and multivariable Cox regression, and four lncRNAs related to prognosis of osteosarcoma were identified and selected for the study. In addition, we constructed a four-lncRNA signature that could act as an independent prognostic factor for use in predicting overall survival in patients with osteosarcoma. Altogether, the current study presents a novel and effective method for the prognosis of patients with osteosarcoma.

## Methods

### Data collection and preprocessing

RNA sequencing (RNA-seq) data on mRNAs and lncRNAs and relevant clinical information of osteosarcoma patients from the TARGET project were downloaded from the website (https://ocg.cancer.gov/programs/target) in June 2021. RNA-seq data files were merged into a matrix file using the merge script of the Perl language (Supplementary Material S1), and the merged RNA matrix is shown in Supplementary Material S2. The gene names were converted from the Ensembl ID to the matrix of the gene symbol through the Ensembl database (http://asia.ensembl.org/index.html). A total of 101 osteosarcoma TARGET RNA-seq data samples were downloaded from the database. The RNA-Seq data were merged with clinical information about the patients, such as vital status, survival time, and gender. There remained 93 samples after excluding those with duplicate data and samples without vital status and survival time. In order to identify survival-related lncRNAs, the 93 samples were divided into two groups according to the vital status. The R package, edgeR (Robinson et al., 2010), was used to identify genes that were differentially expressed between 55 samples from living patients and 38 samples from deceased patients, with a false discovery rate (FDR) < 0.05 and log_2_FC (FC) >1 as the threshold.

### Identification of a prognostic lncRNA signature

Univariate Cox proportional hazard regression analysis was performed using the R survival package (Lee, 2019) to screen out potential lncRNAs related to the prognosis of osteosarcoma patients. Only those lncRNAs with *p* < 0.05 were considered candidate prognostic factors of osteosarcoma. Multivariate Cox proportional hazards regression was performed using the R package, “survival” (Lee, 2019), to identify significant prognostic lncRNAs that affect the survival of osteosarcoma patients. In the multivariate Cox model, sex, age, and tumor metastasis status could be covariates but were not considered in our study. We mainly analyzed the 11 lncRNAs identified in the univariate Cox. Lastly, we chose four lncRNAs (CTB-4E7.1, RP11-553A10.1, RP11-24N18.1, and PVRL3-AS1) according to *p*<0.05 and constructed a prognosis signature of osteosarcoma patients, based on the expression level of the four lncRNAs as a variable. The risk score of the prognosis signature was calculated as follows:

$$Risk\;Score\;\left(RS\right)=\sum coefficient\;\left(lncRNAi\right)✖expression\left(lncRNAi\right).$$

Here, *lncRNAi* is the identifier of the selected lncRNAs. A high-risk score indicates poor survival for osteosarcoma patients.

### Survival analysis and ROC evaluation

One essential characteristic of a reasonable prognostic signature is that it should have no correlation with the currently used clinicopathological prognostic factors. In order to evaluate and measure the independence as well as applicability of such a signature, we calculated the prognostic risk score for each sample based on the signature and categorized patients into high-risk and low-risk groups, taking the median value of the risk score into consideration. Kaplan–Meier analysis was also performed to create overall survival curves. Meanwhile, log-rank tests were utilized to evaluate surviving discrepancy of both high-risk and low-risk patient groups. By calculating the area under the curve (AUC) of the receiver operating characteristic (ROC) curve (Liu et al., 2020; Yu et al., 2020), the performance of lncRNA prognostic signature was evaluated. In this section, Kaplan–Meier analysis was performed using the R package, “survival” (Lorent et al., 2014), and the ROC curve calculation was performed using the R package, “survivalROC”(Lorent et al., 2014).

### Functional enrichment analysis

On the basis of univariate and multivariate analyses, there were four prognostic lncRNAs that could be correlated with the prognosis of osteosarcoma patients. LncRNA-correlated protein-coding genes (PCGs) underlie the foundation of the analysis of functional enrichment. The mRNA sequence data of osteosarcoma samples were obtained from the TARGET data matrix. Pearson correlation coefficients among the expression profiles of all four prognostic lncRNAs and PCGs were calculated to distinguish the co-expression relationships between lncRNAs and PCGs. PCGs with the Pearson correlation coefficient >0.40 and *p*<0.001 were regarded as lncRNA-related PCGs. To determine the biological processes and pathways through which the lncRNAs may play their role, we used functional analysis of Gene Ontology (GO) and the Kyoto Encyclopedia of Genes and Genomes (KEGG) (Kanehisa et al., 2021) via the database for both annotation and visualization, as well as the integrated discovery (http://metascape.org/) internet tool. Significant functional categories were defined as those in which *p*<0.05*.* GO and KEGG pathway analysis was performed using the R package, “clusterProfiler” (Yu et al., 2012).

### Protein–protein interaction network construction

In order to explore the interactions between proteins and the most likely target proteins, protein–protein interaction (PPI) analysis of lncRNA-correlated PCGs was performed using the STRING protein database 11.0 (http://string-db.org/). We set the cut-off criterion for the interaction score as >0.7.

### Gene set enrichment analysis

To explore the important functional phenotypes between the high- and low-risk groups based on the four-lncRNAs signature, we performed gene set enrichment analysis (GSEA). Using c2.all.v7.0.symbols.gmt and c5.all.v7.0.symbols.gmt as reference gene sets, enrichment analysis was performed using GSEA software (ver 4.0.3). A nominal *p*<0.05 and a false discovery rate <0.05 were considered statistically significant.

### Cell culture

The human skeletal muscle cell line (HSMC) and osteosarcoma cell line (OS) were obtained from the American Type Culture Collection (ATCC, VA, USA). Cells were incubated at 37°C with 5 % CO_2_ in Dulbecco’s modified Eagle’s medium (DMEM) (HyClone, UT, USA) supplemented with 10% fetal bovine serum (Gibco, CA, USA) and 1 % penicillin–streptomycin (HyClone, UT, USA).

### Tissue specimens

Tumor samples were obtained from 15 osteosarcoma patients who underwent tumorectomy in Shandong Provincial Hospital from January 2020 to July 2022. Inclusion criteria were as follows: all patients had a definite pathological diagnosis of osteosarcoma and underwent tumor biopsy or resection. Exclusion criteria included history of other types of cancer and history of evidence of severe acute or chronic infection or infectious illness. Patients treated previously for osteosarcoma and patients not willing to give consent were also excluded from the study. Skeletal tissues from 10 trauma patients without any tumor were collected as controls. All tissue samples were stored in liquid nitrogen and briefly stored in a −80°C refrigerator when being processed. The clinical characteristics of the 15 osteosarcoma patients are shown in Supplementary File S3.

### RNA extraction and qRT-PCR analysis

RNA extraction and qRT-PCR analysis were carried out as described in our previous study (Zhang et al., 2021a). Total RNA from cultured cells was extracted using SparkZol reagent (SparkJade, Shandong, China), and cDNA was synthesized with the Evo M-MLV RT Premix for qPCR (AG11706, Accurate Biotechnology, Hunan, China). The qRT-PCR analysis was carried out using the SYBR Green Premix Pro Taq HS qPCR Kit (AG11701, Accurate Biotechnology, Hunan, China) as directed by the manufacturer. Primers were synthesized by BioSune Co., Ltd. (Shanghai, China) and the sequences are shown in Table 1. The qRT-PCR was performed to evaluate the expression levels of the four lncRNAs (CTB-4E7.1, RP11-553A10.1, RP11-24N18.1, and PVRL3-AS1) using a LightCycler480 system (Roche Diagnostics, Switzerland). The lncRNAs expression was normalized to ACTB, and the relative expression level was determined by the 2^-∆∆^CT method.

**TABLE 1:** Specific primer sequences for qRT-PCR.

Primer name	Primer sequence (5′-3′)	Length (bp)
Homo-CTB-4E7.1	F: 5′ CTA​GCC​CAG​CAA​ATG​TGT​CG 3′	124
	R: 5′ TCT​CCC​CTA​CTC​TGC​CTT​ACC 3′	
Homo-RP11-553A10.1	F: 5′ CCG​GCA​AAA​CCA​GAG​TTG​AG 3′	180
	R: 5′ CTG​TGG​GTT​TTG​GCT​TGA​GG 3′	
Homo-RP11-24N18.1	F: 5′ TCC​AGC​GGG​GGA​ATA​GAT​GA 3′	125
	R: 5′ AGT​ATA​TCC​CTC​TGC​CTC​ACA 3′	
Homo-PVRL3-AS1	F: 5′ AAG​AGC​TGA​AAG​CCT​GCA​AAC 3′	175
	R: 5′ CCC​TTT​GTG​ATT​CGT​GCC​TG 3′	
ACTB	F: 5′ AGT​TGC​GTT​ACA​CCC​TTT​CTT​G 3′	149
	R: 5′ CAC​CTT​CAC​CGT​TCC​AGT​TTT 3′	

F, forward; R, reverse.

**FIGURE 1:**
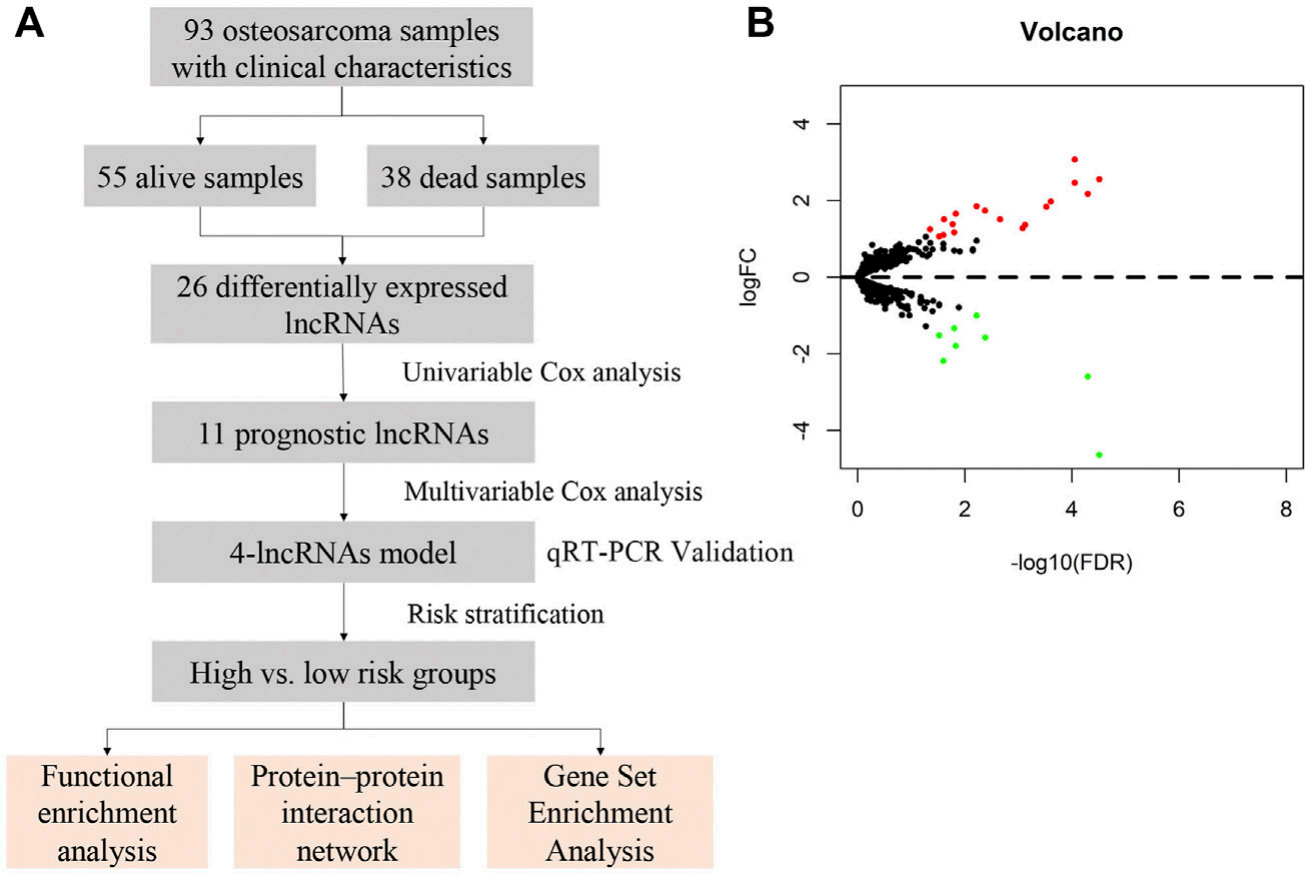
Study design and expression profiles of the 26 survival-related lncRNAs in osteosarcoma. **(A)** Workflow of the present study. **(B)** Volcano plot of differentially expressed lncRNAs in samples from deceased and living patients. Red indicates upregulated lncRNAs in deceased patient samples, and green indicates down-regulated lncRNAs. False discovery rate, FDR<0.05 and logFC>1.

The study design and workflow of the present research is shown in Figure 1A. This study was approved by the Ethics Committee of Shandong Provincial Hospital (the ethics approval number is SZZJJ:NO.2021-419) and was carried out in accordance with relevant guidelines and regulations.

## Results

### Sample characteristics

The RNA-Seq data were obtained from the TARGET-OS database and merged with patient clinical information, such as vital status, survival time, and gender. There were a total of 93 samples including 55 from living patients and 38 from deceased patients, after excluding duplicate data and samples without vital status and survival time. The clinical characteristics are presented in Table 2. The specific clinical characteristics of each sample are shown in Supplementary File S4.

**TABLE 2:** Clinical characteristics of patients with osteosarcoma.

Characteristic	Sample size	Ratio (%)
Sex		
Female	40	43.1
Male	53	56.9
Age (year)		
≤18	73	78.5
>18	20	21.5
Primary tumor site		
Arm/hand	7	7.5
Leg/foot	81	87.1
Pelvis	4	4.3
Other	1	1.1
Disease state at diagnosis		
Metastatic	23	24.7
Non-metastatic	70	75.3
Metastatic site		
Lung	17	18.3
Bone	1	1.1
Lung and bone	5	5.4

### Identification of survival-related, differentially expressed lncRNAs

Differential gene expression between deceased and living patients was compared with the edgeR package using R studio. In total, 26 differentially expressed lncRNAs were identified, of which 18 were upregulated and 8 were downregulated in the deceased group, with thresholds for FDR <0.05 and log_2_FC (FC) >1 (Supplementary File S5). Volcano plot analysis was used to show variations in lncRNA expression between samples form living and deceased patients (Figure 1B).

### Construction of an lncRNA-based multivariable Cox model

To identify prognostic lncRNAs, expression data of 26 differentially expressed lncRNAs and patients’ survival data were subjected to univariate Cox proportional hazards regression analysis. Eleven lncRNAs were found to be significantly associated with overall survival (*p<0.05*) (Supplementary File S6). Multivariate Cox proportional hazards regression analysis was next used to select the optimal lncRNAs as significant independent prognostic factors. Lastly, we identified four lncRNAs (CTB-4E7.1, RP11-553A10.1, RP11-24N18.1, and PVRL3-AS1) according to *p<0.05* and constructed a prognosis signature of osteosarcoma, based on the expression levels of these four lncRNAs. Details about the four lncRNAs are shown in Table 3. The expression of CTB-4E7.1 and RP11-24N18.1 was upregulated and the expression of RP11-553A10.1 and PVRL3-AS1 was down-regulated in deceased compared with living patients. The results of the four prognostic lncRNAs in the univariable and multivariable Cox regression models are shown in Table 4. A four-lncRNA survival-related risk signature for osteosarcoma survival prediction was constructed. This signature was developed as a linear combination of the expression levels of the four lncRNAs weighted by their relative regression coefficients by multivariate Cox regression as follows:

$$Risk\;Score\;\left(RS\right)=\sum coefficient\;\left(lncRNAi\right)✖\;expression\left(lncRNAi\right).$$

**TABLE 3:** Four differentially expressed lncRNAs in deceased osteosarcoma patients compared with living patients.

lncRNA name	Ensembl GENCODE	Fold change (FC)	Log_2_FC	*p-*value	FDR
CTB-4E7.1	ENSG00000253980.1_3	3.327394	1.734393	7.70E-05	0.004176
RP11-553A10.1	ENSG00000206532.2_3	0.288589	-1.79291	0.000458	0.014684
RP11-24N18.1	ENSG00000260570.1_3	2.611741	1.385012	0.000623	0.016891
PVRL3-AS1	ENSG00000242242.5_3	0.349367	-1.51719	0.001483	0.030045

FDR, false discovery rate.

**TABLE 4:** Results of four prognostic lncRNAs in univariable and multivariable Cox regression models.

lncRNA name	Univariable Cox regression			Multivariable Cox regression			
	HR	z	*p*-value	Coefficient	HR	z	*p*-value
CTB-4E7.1	1.32	2.8	5.13E-03	0.25	1.29	2.47	1.34E-02
RP11-553A10.1	0.72	−3.22	1.28E-03	−1.07	0.34	−3.08	2.10E-03
RP11-24N18.1	1.38	3.1	1.94E-03	0.28	1.33	2.78	5.41E-03
PVRL3-AS1	0.75	−2.49	1.28E-02	1.05	2.86	2.43	1.52E-02

HR, hazard ratio.

Based on our results, the formula was:

$$\begin{array}{l} Risk\;Score\;\left(RS\right)=0.25\;✖\;expression\;\left(CTB-4E7.1\right)\\ -1.07✖\;expression\;\left(RP11-553A10.1\right)\\ +0.28✖\;expression\left(RP11-24N18.1\right)\\ +1.05✖\;expression\left(PVRL3-AS1\right). \end{array}$$

### Osteosarcoma survival prediction using the four-lncRNA risk signature

**FIGURE 2:**
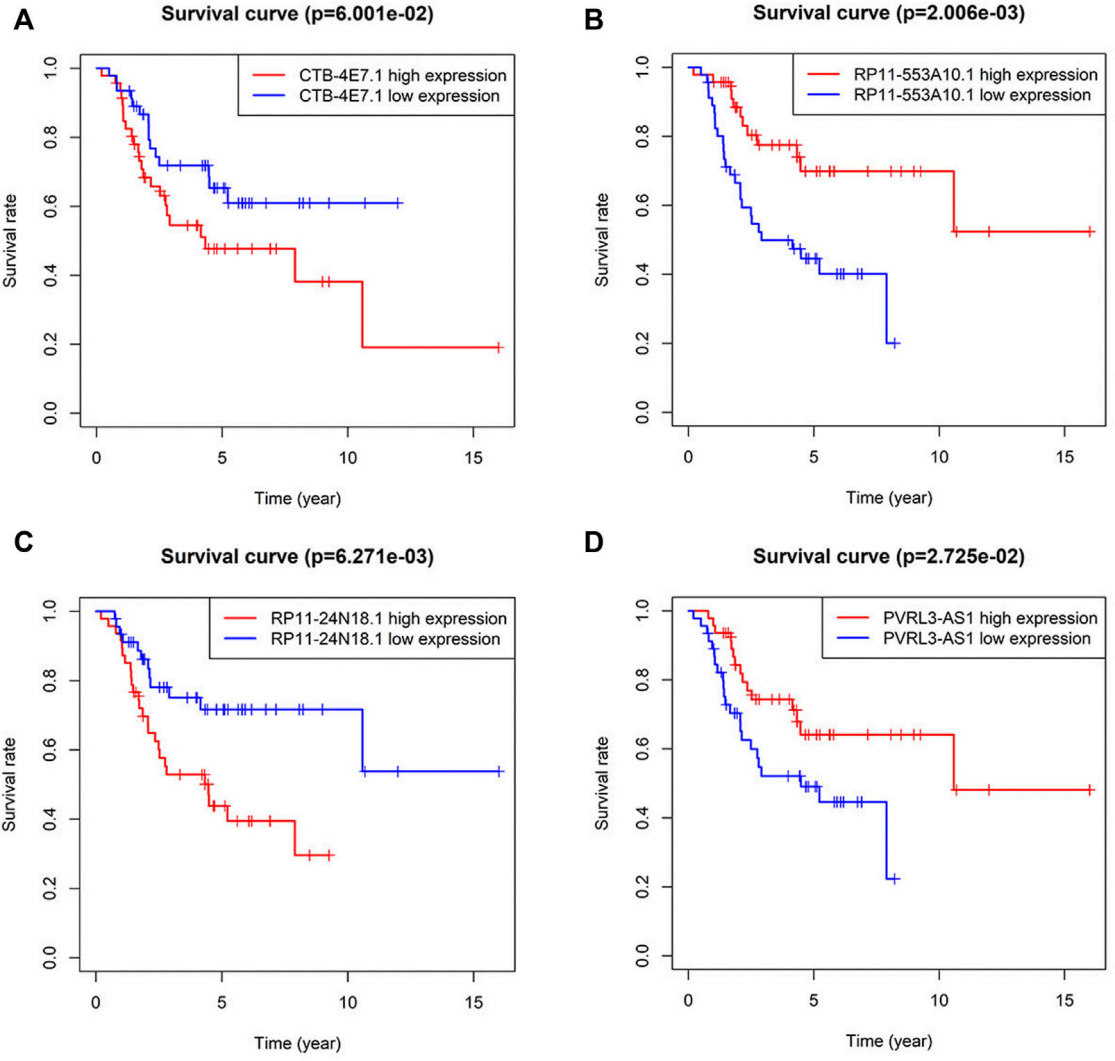
Kaplan–Meier curves for survival of osteosarcoma patients stratified by the four lncRNAs. **(A)** CTB-4E7.1; **(B)** RP11-553A10.1; **(C)** RP11-24N18.1; and **(D)** PVRL3-AS1.

**FIGURE 3:**
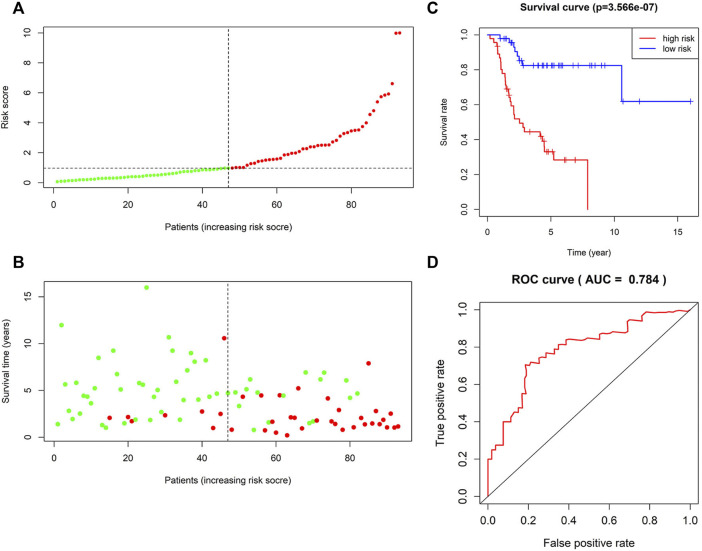
Survival prediction of the four-lncRNA risk signature for osteosarcoma. **(A)** Risk score distribution. Green points represent low-risk patients, and red points represent high-risk patients. **(B)** Distribution of survival time and survival status of patients in high-risk and low-risk groups according to the signature. Green points represent living patients, and red points represent deceased patients. **(C)** Kaplan–Meier curves of the overall survival of high-risk and low-risk osteosarcoma patients based on the four-lncRNA signature. **(D)** Receiver operating characteristic (ROC) curve analysis of the four-lncRNA signature for predicting 5-year survival of patients with osteosarcoma.

Kaplan–Meier analysis of the four lncRNAs was performed and the expression of CTB-4E7.1 showed no influence on patient survival (*p*>0.05) (Figure 2A). The expression of RP11-553A10.1 and PVRL3-AS1 were positively correlated with survival of osteosarcoma patients, while RP11-24N18.1 showed the opposite effect (Figures 2B–D). Next, we calculated the prognostic risk score for each sample according to this prognosis signature, and the median value of the risk score was 0.975 (Supplementary File S7). Then we divided patients into high-risk and low-risk groups according to the median value of the risk score (Figure 3A). The survival status and survival time of the osteosarcoma patients is shown in the dot plot (Figure 3B), and indicates that as the risk score increased, the risk of death also increased. The specific survival times associated with samples from the deceased and the length of follow-up for the living patient samples are shown in Supplementary File S4. Kaplan–Meier analysis was performed to compare the survival rates between the high-risk and low-risk groups. The overall survival of the two groups differed significantly, suggesting that the survival rate of high-risk patients was lower than that in low-risk patients at any point (*p* = 3.566e-07). The overall five-year survival in the high-risk and low-risk groups was 33.1% and 82.5%, respectively (Figure 3C). In addition, we constructed a ROC curve to assess the predictive performance of the four-lncRNA prognostic risk model (Figure 3D). The AUC value was 0.784, proving that the prognostic signature was convincing and had the potential to evaluate the survival of osteosarcoma patients.

### Functional enrichment analysis

**FIGURE 4:**
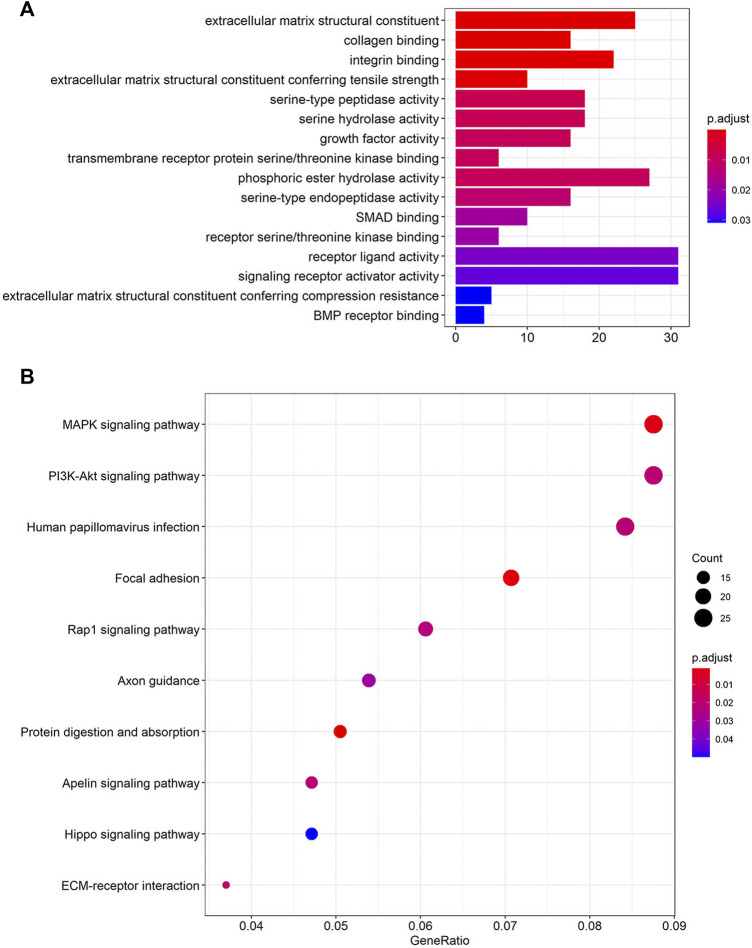
Functional enrichment analysis of the four lncRNAs based on their correlated protein-coding genes (PCGs). **(A)** GO enrichment analysis of PCGs. **(B)** KEGG pathway enrichment analysis of PCGs. GO: Gene Ontology; KEGG: Kyoto Encyclopedia of Genes and Genomes (Lorent et al., 2014; Liu et al., 2020; Kanehisa et al., 2021).

A total of 746 PCGs related to the four lncRNAs (CTB-4E7.1, RP11-553A10.1, RP11-24N18.1, and PVRL3-AS1) with Pearson correlation coefficients >0.40 and *p*<0.001 were identified. To explore the functional implications of the four-lncRNA signature, we performed GO and KEGG functional enrichment analysis to reveal the specific functional categories of lncRNA-correlated PCGs. Figure 4A highlights the most significantly enriched GO terms of the PCGs. The top three were extracellular matrix structural constituents, collagen binding, and integrin binding. After KEGG analysis, we found that the MAPK signaling pathway, the PI3K-Akt signaling pathway, and human papillomavirus infection were the most three enriched pathways (Figure 4B).

### Protein–protein interaction network construction

**FIGURE 5:**
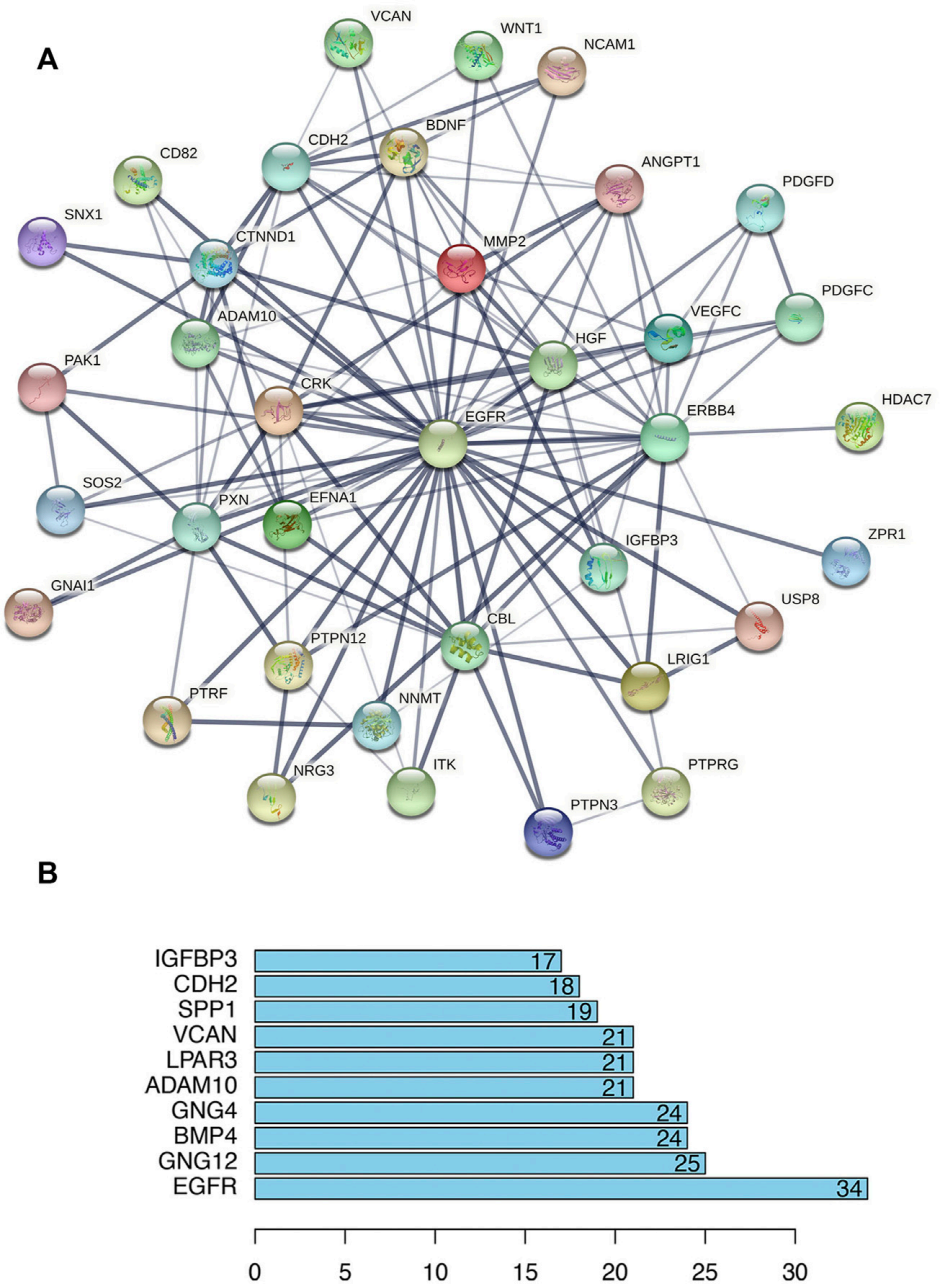
Protein–protein interaction (PPI) analysis of lncRNA-correlated PCGs. **(A)** PPI network centered on *EGFR* (STRING). Nodes represent proteins, and edges represent protein–protein connections. **(B)** Bar plot shows the top ten ranked proteins.

To deduce the connections among the lncRNA-correlated PCGs, a PPI network was constructed using the STRING protein database, including 353 nodes and 739 edges (Supplementary File S8). We calculated the number of genes connected to each node (gene) in the network and the average was 4.19. The top 10 genes with the most connections are shown in Figure 5B and include *EGFR*, *GNG12*, *BMP4*, and *GNG4*, which might play important roles in the PPI network. *EGFR* had the most connections to other genes, and Figure 5A shows the *EGFR* network.

### Gene set enrichment analysis

**FIGURE 6:**
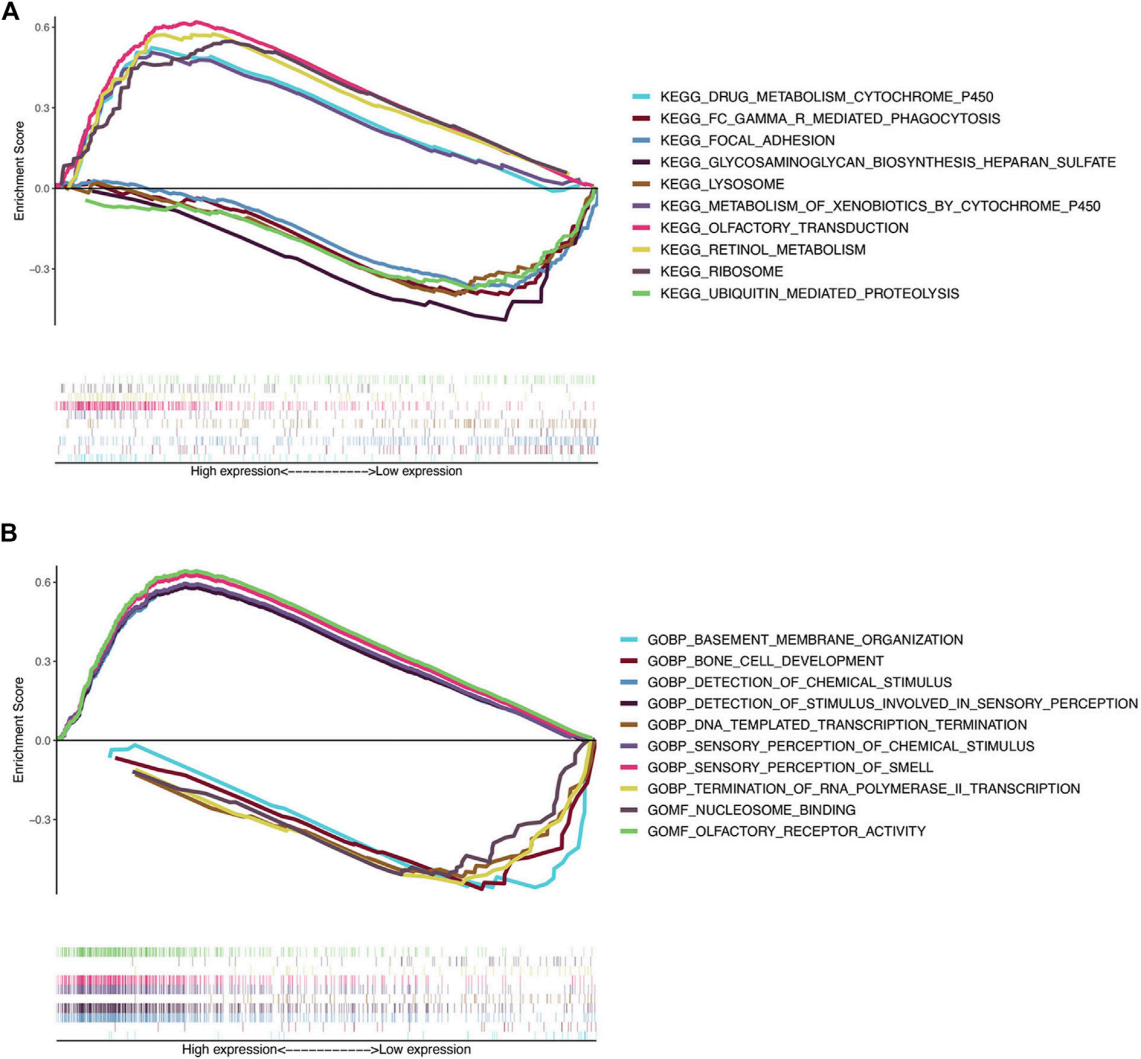
Gene set enrichment analysis. **(A)** Top five KEGG pathways in the high-risk group (above the horizontal line) and in the low-risk group (below the horizontal line). **(B)** Top five GO terms enriched in the high-risk group (above the horizontal line) and in the low-risk group (below the horizontal line).

To further understand the potential biological processes involved and the underlying signaling pathways, GSEA was carried out to determine the associated signaling pathways between the two risk groups. Through KEGG pathway analysis, the top five pathways enriched in the high-risk group were olfactory receptor activity, sensory perception of smell, sensory perception of chemical stimulus, detection of chemical stimuli, and detection of stimuli involved in sensory perception. In contrast, in the low-risk group five other pathways, including DNA templated transcription termination, nucleosome binding, bone cell development, termination of RNA polymerase II transcription, and basement membrane organization were significantly enriched (Table 5 and Figure 6A). GO gene sets analysis showed that the high-risk groups were positively associated with olfactory transduction, ribosomes, retinol metabolism, drug metabolism, cytochrome P450 drug metabolism, and metabolism of xenobiotics by cytochrome P450, and negatively related to lysosomes, focal adhesion, Fc gamma R-mediated phagocytosis, ubiquitin-mediated proteolysis, and glycosaminoglycan biosynthesis heparan sulfate (Table 6 and Figure 6B).

**TABLE 5:** Top five enriched KEGG terms in the high-risk group and in the low-risk group.

KEGG term	ES	NES	*p*-value	FDR q-value
Olfactory receptor activity	0.643	3.541	<0.001	<0.001
Sensory perception of smell	0.628	3.497	<0.001	<0.001
Sensory perception of chemical stimuli	0.595	3.378	<0.001	<0.001
Detection of chemical stimuli	0.586	3.332	<0.001	<0.001
Detection of stimuli involved in sensory perception	0.581	3.287	<0.001	<0.001
DNA templated transcription termination	−0.530	−2.353	<0.001	2.88E-03
Nucleosome binding	−0.526	−2.237	<0.001	2.93E-02
Bone cell development	−0.592	−2.192	<0.001	3.76E-02
Termination of RNA polymerase II transcription	−0.474	−2.188	<0.001	2.89E-02
Basement membrane organization	−0.456	−2.174	<0.001	2.80E-02

ES, enrichment score; NES, normalized enrichment score.

**TABLE 6:** Top five enriched GO terms in the high-risk group and in the low-risk group.

GO term	ES	NES	*p*-value	FDR q-value
Olfactory transduction	0.620	3.434	<0.001	<0.001
Ribosomes	0.548	2.485	<0.001	<0.001
Retinol metabolism	0.574	2.449	<0.001	<0.001
Drug metabolism, cytochrome P450 drug metabolism	0.524	2.275	<0.001	<0.001
Metabolism of xenobiotics by cytochrome P450	0.506	2.195	<0.001	1.47E-04
Lysosomes	−0.507	−2.069	<0.001	1.00E-02
Focal adhesion	−0.404	−2.012	<0.001	1.36E-02
Fc gamma R-mediated phagocytosis	−0.374	−2.000	<0.001	9.54E-03
Ubiquitin-mediated proteolysis	−0.404	−1.912	<0.001	1.57E-02
Glycosaminoglycan biosynthesis heparan sulfate	−0.382	−1.905	<0.001	1.26E-02

ES, enrichment score; NES, normalized enrichment score.

### Validation of lncRNA expression

**FIGURE 7:**
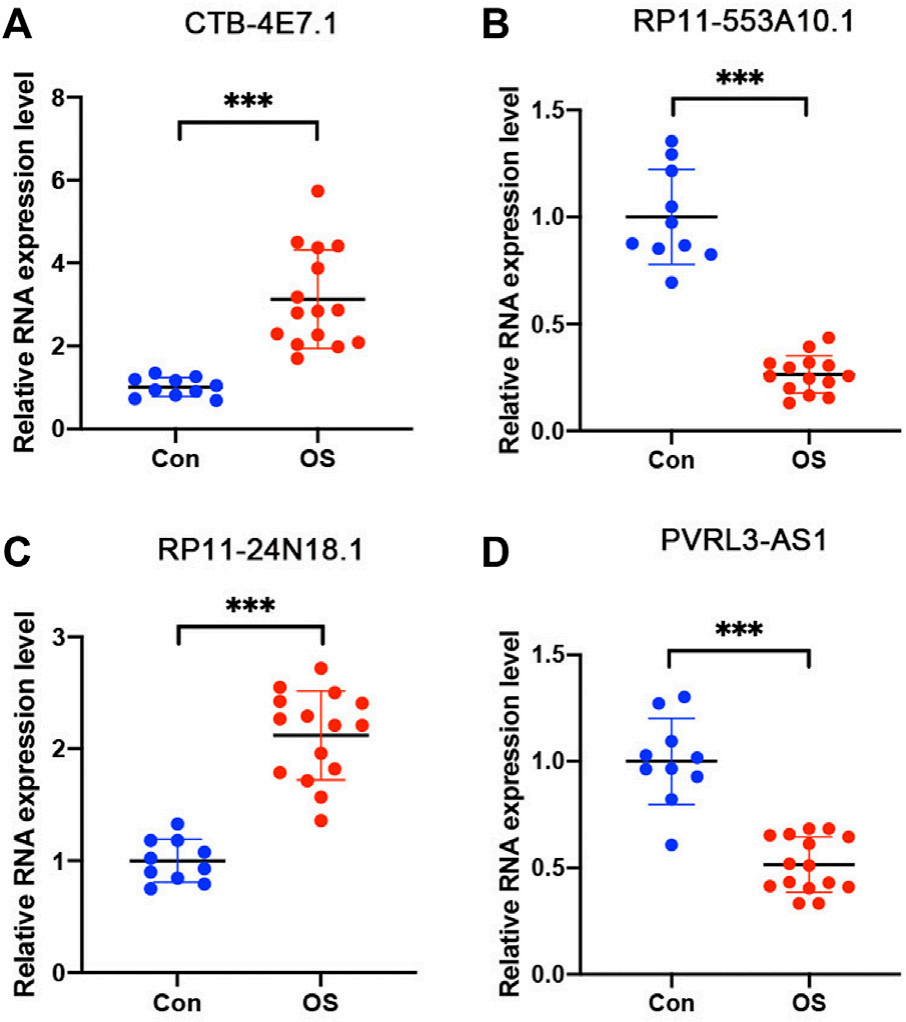
Expression level of the four lncRNAs validated by RT-qPCR in osteosarcoma tissues. **(A)** CTB-4E7.1; **(B)** RP11-553A10.1; **(C)** RP11-24N18.1; and **(D)** PVRL3-AS1. Control (CON) is human skeletal muscle from trauma patients (*n* = 10); OS, osteosarcoma tissues (*n* = 15). Data are mean ± SD. *p<0.05, **p<0.01, ***p<0.001 (Student’s *t*-test).

**FIGURE 8:**
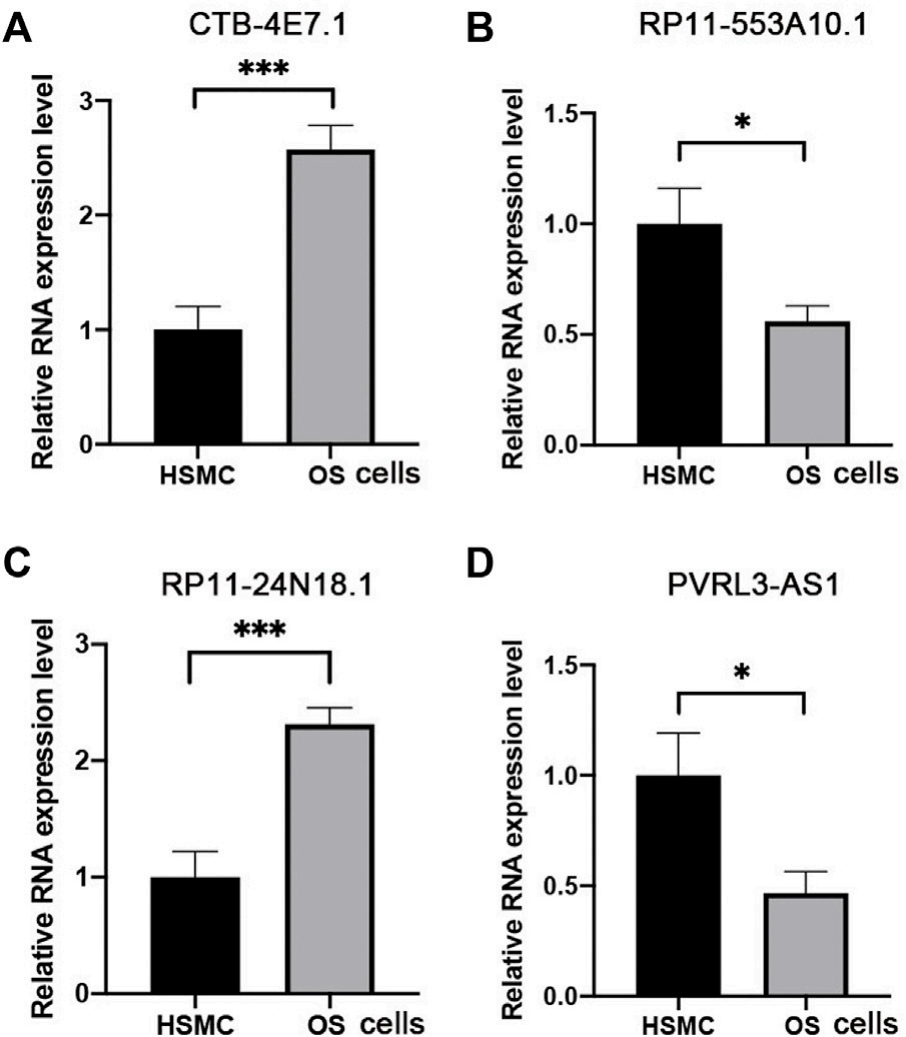
Expression level of the four lncRNAs validated by RT-qPCR in osteosarcoma cells. **(A)** CTB-4E7.1; **(B)** RP11-553A10.1; **(C)** RP11-24N18.1; and **(D)** PVRL3-AS1. HSMC, human skeletal muscle cell; OS cells, osteosarcoma cells. Data are mean ± SD (*n* = 3 biologically independent samples). *p<0.05, **p<0.01, ***p<0.001 (Student’s *t*-test).

The expression of the four selected lncRNAs (CTB-4E7.1, RP11-553A10.1, RP11-24N18.1, and PVRL3-AS1) was validated by RT-qPCR in osteosarcoma tumors. Skeletal muscle from trauma patients served as the control tissue. The results showed that CTB-4E7.1 and RP11-24N18.1 were upregulated in osteosarcoma tissues, while RP11-553A10.1 and PVRL3-AS1 were down-regulated in osteosarcoma compared with controls. (Figure 7). In addition, we detected the expression of the four lncRNAs in osteosarcoma cells compared with human skeletal muscle cell (HSMC) by RT-qPCR. The results were consistent with the clinical samples (Figure 8).

## Discussion

Osteosarcoma is the most common primary malignant tumor of bone, with a poor prognosis and a high incidence rate in children and adolescents. Research has shown that a series of clinical characteristics, such as tumor size, localized sites, stage at presentation, and response to chemotherapy are significant for predicting overall survival of osteosarcoma patients; however, the lack of specific and sensitive biomarkers of prognosis is still an urgent problem in the management of osteosarcoma patients. In recent years, with the development of microarray and high-throughput sequencing technology, the expression patterns of mRNAs and lncRNAs have been widely studied. Many mRNAs have been identified as prognostic markers for osteosarcoma patients, and several prognostic signatures based on mRNAs have been constructed (Zhang et al., 2020a; Rothzerg et al., 2021; Wu et al., 2021; Yin et al., 2021). Although several previous studies have identified a series of lncRNAs that play an important role in osteosarcoma, proof of the efficacy of lncRNAs as prognostic markers of osteosarcoma is still limited. SiYuan Yu et al. identified a five-lncRNA, metastasis-related risk signature for osteosarcoma survival prediction, and the AUC for predicting 5-year survival was 0.745 (Yu et al., 2021). Gao et al. (2020) constructed a multivariable Cox regression model based on seven lncRNAs for prognosis prediction, among which AC011442.1 was assumed to act as an oncogenic driver in osteosarcoma. Our study identified a four-lncRNAs risk signature for osteosarcoma survival prediction.

In this study, we first found 26 survival-related differentially expressed lncRNAs between deceased and living patients based on RNA sequencing data and clinical information obtained from the TARGET database. These 26 differentially expressed lncRNAs and the patient survival data were next subjected to univariate and multivariate Cox proportional hazards regression, and four lncRNAs (CTB-4E7.1, RP11-553A10.1, RP11-24N18.1, and PVRL3-AS1) were identified. A four-lncRNA risk signature for osteosarcoma survival prediction was constructed and the subsequent log-rank tests and Kaplan–Meier analyses further confirmed the predictive value of the risk score. A high-risk score was related to poor prognosis. Based on the risk scores, we divided the patients into a high-risk group (>0.975) and a low-risk group (<0.975), and then calculated the five-year survival rate by Kaplan–Meier analysis. In comparison with other signatures, our AUC value of 0.784 was larger than the 0.745 AUC value of SiYuan Yu’s signature (Yu et al., 2021). This confirmed that our prognostic signature was convincing and had the potential to evaluate the survival of patients with osteosarcoma.

To explore the functional implication of the four-lncRNAs signature, we performed GO and KEGG functional enrichment analysis to reveal particular functional categories of lncRNA-correlated PCGs. The most highly enriched GO terms were extracellular matrix structural constituents, collagen binding, and integrin binding. Collagen and integrin are important constituents of the extracellular matrix (Zeltz and Gullberg, 2016). The extracellular matrix in the tumor microenvironment is a complex network of structural and instructional molecules tightly connected to the tumor cells, and the disorganization of extracellular matrix structure promotes tumor cellular transformation and metastasis (Jain et al., 2013; Schaefer and Reinhardt, 2016). The extracellular matrix microenvironment plays an important role in regulating tumor cell behavior, and many studies have proved that the extracellular matrix undergoes numerous changes in composition and organization in cancer development and progression (Crotti et al., 2017). Based on this and our GO enrichment results, we speculated that the four-lncRNAs signature might reflect the risk of osteosarcoma progression by regulating the extracellular matrix and tumor microenvironment.

After KEGG analysis, the MAPK signaling pathway, PI3K-Akt signaling pathway, and human papillomavirus infection were the most highly enriched pathways. As two well-studied pathways, the MAPK and PI3K-Akt signaling pathways have been proved to play important roles in osteosarcoma, by regulating cell proliferation, apoptosis, migration, and metastasis (Cheng et al., 2017; Han et al., 2019; Zhang et al., 2020b; Zhang et al., 2020c). Thus, our four-lncRNAs signature might influence osteosarcoma prognosis through effects on the MAPK and PI3K-Akt signaling pathways, which supports the credibility of the signature from its functional linkage. Interestingly, the third most enriched pathway had to do with human papillomavirus infection. Papillomavirus infection is a critical factor in the development of cervical cancer in women. However, a relationship between papillomavirus infection and osteosarcoma is still not clear, and this finding points the way to a new direction in the study of osteosarcoma etiology.

We created a PPI network to identify the potential biological interactions among co-expressed lncRNA-associated PCGs (Quo et al., 2012; Li et al., 2019; Chen et al., 2020). The results showed that *EGFR*, *GNG12*, *BMP4*, and *GNG4* were key players in the PPI network. Several studies have shown a link between *EGFR* and *BMP4* in osteosarcoma (Li et al., 2020a; Nakajima et al., 2021). However, a role for *GNG12* and *GNG4* in the mechanism of osteosarcoma progression has not been reported, which also provides a new direction for osteosarcoma research.

In further investigating the lncRNA signature, we carried out GSEA to determine the associated signaling pathways between high-risk and low-risk groups. The most enriched pathways in the high-risk group were in olfactory receptor activity and the most enriched GO term was olfactory transduction in the high-risk group. What is the relationship between olfactory function and osteosarcoma? This result is intriguing. Olfactory receptor activity and olfactory transduction have been found to be involved in the regulation of cell-cell recognition, migration, proliferation, the apoptotic cycle, exocytosis, and pathfinding processes. Additionally, accumulating evidence showed that olfactory receptors were highly expressed in different cancer tissues and they have the potential to serve as diagnostic and therapeutic targets (Massberg and Hatt, 2018). However, the role of olfactory receptor activity and olfactory transduction in osteosarcoma has not been studied and needs further investigation.

Lastly, we verified the expression of the four lncRNAs in clinical samples and osteosarcoma cell lines, since these four lncRNAs have never been reported, and they are only listed in the database. We validated their authenticity by qRT-PCR on clinical samples and cell lines.

The potential biological mechanisms of the four identified biomarkers for prognosis prediction include a number of possibilities. Based on the GO enrichment results, the four-lncRNAs signature might influence osteosarcoma by regulating the extracellular matrix and tumor microenvironment (Cohen et al., 2016; Zhou et al., 2016; Yan et al., 2018; Zheng et al., 2019), while the KEGG data suggested that MAPK and PI3K-Akt signaling may be important through their effects on cell proliferation, apoptosis, migration, and metastasis (Xiang et al., 2014; Li et al., 2015; Zi et al., 2015; Li et al., 2020b; Yang et al., 2020; Naz et al., 2021). Olfactory receptor activity and the olfactory transduction pathway may participate in the progression of osteosarcoma by RNA and DNA modifications (Wang et al., 2015; Jin et al., 2020; Wang et al., 2020). In the future, we will explore the mechanism of the four lncRNAs through cell and animal experiments, and the causal effects of these our lncRNAs on the prognosis will be further investigated through examination of additional multi-omics data sets (Hou et al., 2020; Wu et al., 2020; Wang et al., 2021; Zhang et al., 2021b).

The signature presented in this study has several advantages over previous prognostic models. First, the four lncRNAs were selected from survival-related lncRNAs, which were differently expressed in living vs. deceased patients. The other studies mainly selected metastasis-related or immune-related genes. The survival-related lncRNAs were better able to predict osteosarcoma prognosis. Compared with other signatures, our AUC value (0.784) was larger. These facts demonstrated that our prognostic signature was most convincing and had the potential to evaluate the survival of patients with osteosarcoma. In addition, the functional analysis of the signature indicated that the lncRNAs might influence osteosarcoma by regulating the extracellular matrix, the tumor microenvironment, or the MAPK and PI3K-Akt signaling pathways.

Some limitations of this study should be pointed out, however. First, the dysregulated survival-related lncRNAs in osteosarcoma were analyzed using previously released data sets and validated by RT-qPCR in osteosarcoma tissues and cells, but functional verification was still lacking. Future experimental validation is needed to verify our findings. Second, all the data in our research were collected from the TARGET database, and an external validation cohort for the prognostic model is needed. The TARGET database is dedicated to comprehensive genetic information on childhood tumors, like osteosarcoma. The data in the TARGET database include mRNA, miRNA, lncRNA, and detailed clinical information, and in our study, we used lncRNA-seq data, mRNA-seq data, and clinical information. For this reason, we only used TARGET data and did not include other public data. In the future, we will explore the public data related to osteosarcoma from the GEO and TCGA databases. However, there may be duplications in these databases, so careful screening will be necessary before using data from them. We hope that, when more new datasets become available in the near future, we can further confirm the accuracy and therapeutic efficacy of our signature. In addition, with the rapid development of artificial intelligence (AI), it is being used more and more in clinical medicine and bioinformatics. AI provides new ideas and methods for diagnosis and prognosis prediction of some diseases. In planned studies, we will adapt and apply AI to construct and test new clinical prediction models (Chen et al., 2022; Gao et al., 2022).

## Conclusion

We established a novel risk signature and demonstrated its credibility and rationale for osteosarcoma survival prediction. The signature not only serves as a new biomarker for predicting osteosarcoma prognosis but also provides new insights into the molecular mechanisms of osteosarcoma progression. We will further confirm the signature’s performance using external validation cohorts in the near future. It is hoped that these studies will be applied to the diagnostic and prognostic assessment of osteosarcoma patients, provide new insights into the pathogenesis of osteosarcoma, and promote the development of more effective biomarkers.

## Data Availability

The datasets presented in this study can be found in online repositories. The names of the repository/repositories and accession number(s) can be found in the article/Supplementary Material.
