# Supplementary figures and images for: A four-lncRNA risk signature for prognostic prediction of osteosarcoma
Source: Front Genet. 2023 Jan 4;13:1081478. doi: 10.3389/fgene.2022.1081478 (PMC9847501; doi:10.3389/fgene.2022.1081478)

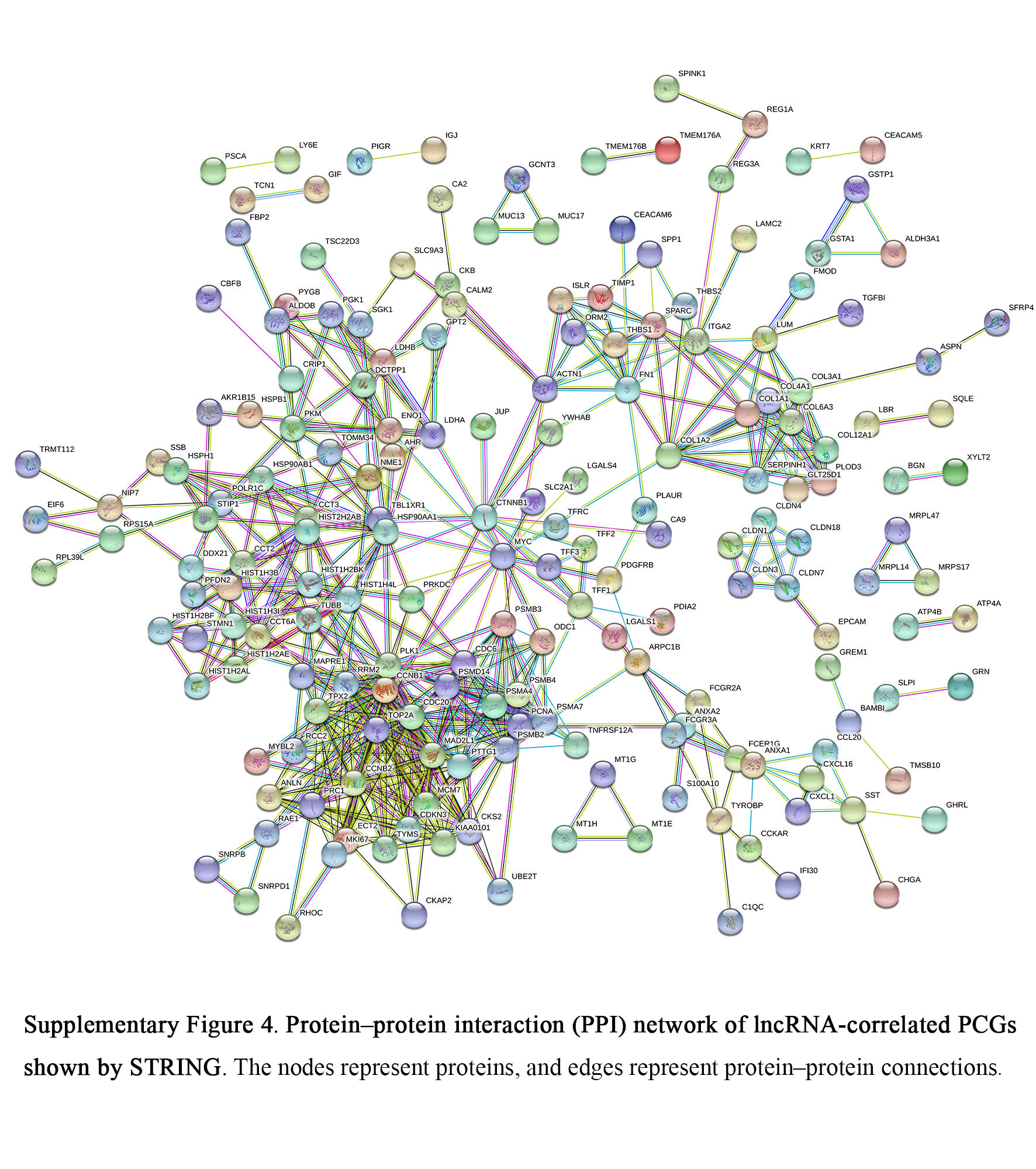

Supplement: Supplementary file 1 [file Image1.JPEG]
